# Comprehensive Evaluation of Storage Stability and Cytotoxicity of Co-Spray-Dried Theophylline Dry Powders for Inhalation: Follow-Up Study

**DOI:** 10.3390/pharmaceutics18081027

**Published:** 2026-08-19

**Authors:** Lomass Soliman, Dóra Paróczai, Katalin Burián, Rita Ambrus

**Affiliations:** 1 Institute of Pharmaceutical Technology and Regulatory Affairs, Faculty of Pharmacy, University of Szeged, Eötvös utca 6, H-6720 Szeged, Hungary; lomass.soliman@szte.hu; 2 Department of Medical Microbiology and Immunobiology, University of Szeged, Dóm Square 10, H-6720 Szeged, Hungary; paroczai.dora@med.u-szeged.hu (D.P.); burian.katalin@med.u-szeged.hu (K.B.); 3 Department of Pulmonology, Albert Szent-Györgyi Medical School, University of Szeged, Tisza Lajos krt 107, H-6720 Szeged, Hungary

**Keywords:** A549 cell line, Andersen cascade impactor, co-spray-drying, dry powder inhaler, leucine, raffinose, recrystallization, stability, theophylline, trehalose

## Abstract

**Background/Objectives**: The stability and biological safety of newly developed formulations must be established to support their therapeutic efficacy and clinical translation in pulmonary drug delivery. Therefore, this follow-up study comprehensively evaluated the short- and long-term stability and the in vitro cytotoxicity of optimized, co-spray-dried theophylline (THN) dry powders for inhalation against A549 lung epithelial cells. **Methods**: Two established formulations were selected: THN-RAF (raffinose–leucine–glycine based) and THN-TRE (trehalose–leucine based). Stability was assessed under accelerated conditions (40 °C/75% RH, 3 months) and long-term desiccator storage (25 °C, 1 year) using laser diffraction, SEM, XRPD, FTIR, DSC, TGA, and Andersen Cascade Impaction. As THN-TRE had been previously confirmed to be cytocompatible, only THN-RAF and its components were evaluated against A549 human alveolar epithelial cells using the MTT assay. **Results:** Under accelerated conditions, both formulations exhibited pronounced recrystallization (*X*c up to 89.9%), agglomeration (D [0.9] up to 217.08 µm for THN-TRE), and deterioration in aerodynamic performance (FPF as low as 11.55%, MMAD up to 6.68 µm). By contrast, long-term desiccator storage induced substantial recrystallization (*X*c up to 80.7%) while preserving thermal, chemical, and aerodynamic performance (FPF ≈ 40%; MMAD 4.99–5.21 µm). THN-RAF was more resistant to stress-induced agglomeration than THN-TRE. Cytotoxicity assessment confirmed cytocompatibility of THN-RAF, with cell viability exceeding 70.99% at all tested concentrations (up to 500 µg/mL). **Conclusions:** These findings reveal a marked discrepancy between the outcomes of ICH accelerated testing and long-term desiccator storage. They underscore the importance of considering moisture-protective packaging configurations when designing stability protocols for amorphous inhalable formulations.

## 1. Introduction

Theophylline (THN) is a methylxanthine bronchodilator that has long been used in the management of asthma and chronic obstructive pulmonary disease (COPD) due to its bronchodilator and anti-inflammatory properties [1]. Its pharmacological effects are mediated through multiple mechanisms, including non-selective phosphodiesterase inhibition, adenosine receptor antagonism, and restoration of histone deacetylase-2 activity at lower concentrations, which may enhance corticosteroid responsiveness [1,2]. Low-dose theophylline has also been investigated as an adjunctive anti-inflammatory therapy, although recent clinical studies have reported variable therapeutic benefits [3]. Consequently, pulmonary delivery remains an attractive strategy to maximize local drug concentrations while minimizing systemic exposure and associated adverse effects [4]. However, despite its therapeutic potential, no inhaled theophylline product is currently available for routine clinical use [4].

Pulmonary drug delivery is constrained by several anatomical, physiological, and immunological barriers [5,6,7]. Nevertheless, inhalation offers a non-invasive, targeted route with the potential for rapid absorption. Other challenges include low pulmonary deposition efficiency, inadequate drug retention, poor patient adherence, and incorrect inhaler technique [5,8]. Formulation-related concerns include drug stability, particle size distribution, and excipient safety [9]. Several devices are available for direct pulmonary administration, including dry powder inhalers (DPIs).

DPIs can provide high physicochemical stability and efficiently deliver relatively high drug doses to the lungs [10]. They are also environmentally preferable [11], and offer a compact, portable, and coordination-free mode of administration; however, their performance depends on the patient generating sufficient inspiratory flow [12]. DPIs are generally easy to use and are associated with high patient adherence [13]. They are compatible with diverse active ingredients, including small molecules and biologics [7,14]. Recent technological advances have sought to improve DPI efficacy, dose uniformity, and physical stability [15], including through spray drying [16]. Co-spray-drying is an advanced particle-engineering approach that can improve the aerosolization and physicochemical stability of DPI formulations compared with conventional systems [17]. Co-spray-dried fine excipients have also been reported to improve dispersibility by filling carrier macropores [18]. When integrated with quality-by-design principles, spray drying can generate stable powders with optimized particle attributes for efficient pulmonary delivery and controlled release [19].

Nevertheless, the stability of newly developed co-spray-dried DPIs remains insufficiently characterized. Regulatory guidance, particularly the International Council for Harmonization of Technical Requirements for Pharmaceuticals for Human Use (ICH) Q1A–Q1F guidelines, defines protocols for pharmaceutical stability testing [20,21]. The physical stability of inhalable dry powders is critical because solid-state changes may impair aerosolization performance [22]. Amorphous saccharide-based powders are particularly challenging because they may recrystallize during storage, thereby reducing aerodynamic performance [23]. These risks can be mitigated through moisture-protective packaging, hydrophobic excipient coatings such as leucine or stearates, and formulations engineered to have high glass-transition temperatures (Tg) [23]. Appropriate analytical methods are likewise essential for detecting stability-related changes [24].

For capsule-based DPIs, capsule composition (e.g., gelatin or hydroxypropyl methylcellulose [HPMC]) affects moisture interactions and electrostatic charging and therefore influences powder emission, retention, aerosolization efficiency, puncture behavior, and formulation stability [25,26]. Our group previously found that HPMC capsules provided the most favorable conditions for powder stability. By maintaining an appropriate residual moisture content and structural integrity while minimizing fragmentation after activation, these capsules supported superior structural and aerodynamic stability [27]. We have also investigated the physicochemical and aerodynamic stability of several novel spray-dried inhalation powders [28,29,30].

As pulmonary administration requires a favorable safety profile, in vitro cytotoxicity assessment is an essential step in predicting respiratory toxicity and identifying potential safety concerns before animal and clinical studies [31,32]. The A549 human alveolar epithelial adenocarcinoma cell line is widely accepted for evaluating the cytotoxicity of DPI formulations and has demonstrated good predictive value in previous studies [33,34,35].

Consequently, the present work builds upon our previously developed and optimized DPI formulations [33,36]. Co-spray-drying THN with saccharide–amino acid carriers—trehalose dihydrate (TRE) or raffinose pentahydrate (RAF) combined with leucine (LEU) or a leucine–glycine mixture (LEU–GLY)—yielded partially amorphous microparticles with markedly improved solubility, rapid THN dissolution, fine particle fractions (FPFs) of 43–48%, and predicted deep lung deposition of 36–40%. These carrier systems may enhance therapeutic efficacy while enabling dose reduction.

Although our previous studies established the formulation design and initial characterization of THN-TRE and THN-RAF DPI systems, their storage stability remained unexplored. The present study extends this earlier work by systematically evaluating the long-term and accelerated stability of both optimized formulations, with emphasis on physicochemical, solid-state, thermal, and aerodynamic performance after storage. Furthermore, the in vitro cytocompatibility of THN-RAF was evaluated using A549 cells to establish its preliminary pulmonary safety profile, complementing the previously reported safety assessment of THN-TRE [33]. This follow-up investigation provides the stability evidence required to assess their suitability for further pharmaceutical development. To the best of our knowledge, this is the first report presenting up to 1 year of stability data for these specific saccharide-based DPI formulations in combination with cytotoxicity profiling, thereby addressing an important gap in the literature.

## 2. Materials and Methods

### 2.1. Materials

Anhydrous theophylline (THN), used as the active ingredient, was supplied by Hungaropharma Ltd. (Budapest, Hungary). The excipients were raffinose pentahydrate (RAF; Tokyo Chemical Industry Co., Ltd., Tokyo, Japan), trehalose dihydrate (TRE; Sigma-Aldrich Chemie GmbH, Steinheim, Germany), L-leucine (LEU; Molar Chemicals Kft., Budapest, Hungary), and glycine (GLY; VWR International LLC, Leuven, Belgium). Ezeeflo™ size 3 hydroxypropyl methylcellulose (HPMC) capsules (ACG-Associated Capsules Pvt. Ltd., Mumbai, India) were used to contain the powders during storage. Soft multilayer polyethylene bags (40–100 µm thick) were purchased from Patika Pack Kft. (Budapest, Hungary). Potassium bromide (J&K Scientific Limited, Beijing, China) was used for FTIR background measurements. Distilled water was obtained using an in-house Millipore Direct-Q 5 UV water-purification system (Millipore, Molsheim, France). A mixture of sorbitan monooleate (Span^®^ 80; Sigma-Aldrich Chemie GmbH, Steinheim, Germany) and cyclohexane (VWR BDH Chemicals, Paris, France) was used to coat the aerodynamic-impactor collection plates. Aliquots were filtered through 0.45 µm Millex-HV syringe filters (Millipore Corporation, Bedford, MA, USA). [3-(4,5-dimethylthiazol-2-yl)-2,5-diphenyltetrazolium bromide] and sodium dodecyl sulfate were supplied by (Sigma, St. Louis, MO, USA).

### 2.2. Preparation of the Optimized Tested Formulations

Saccharide-based co-spray-dried carrier systems containing amino acids were previously optimized through systematic formulation studies to improve production yield, morphology, aerodynamic performance, and drug loading. Two saccharides (RAF and TRE) and three amino acids (LEU, GLY, and L-arginine) were screened. Based on these investigations, two optimized formulations, THN-TRE (TRE-LEU-based) [33] and THN-RAF (RAF-LEU-GLY-based) [36] were selected for further study.

Fresh samples were prepared from 200 mL stock solutions containing THN and the optimized carriers in 10% (*v*/*v*) ethanol, which was used because of the limited aqueous solubility of THN. The solid components were dissolved by ultrasonic sonication (Elmasonic S30H; Schmidbauer GmbH, Singen, Germany), followed by magnetic stirring at a controlled temperature to ensure complete dissolution (AREC.X; Velp Scientifica Srl, Usmate Velate, Italy). THN-containing microparticles were produced using a Büchi B-191 mini spray dryer (Büchi, Flawil, Switzerland) at an inlet temperature of 130 °C and an outlet temperature of approximately 80 °C, with a feed-pump rate of 5%, an aspirator rate of 75%, and a gas-flow rate of 600 L/h. The powders were collected in sealed glass vials and stored in a desiccator to minimize moisture uptake before and during analysis of the fresh samples [33,36]. Results obtained at this time point served as the baseline for the stability studies (hereafter, 0 mon).

A single spray-dried batch was produced for each formulation and aliquots of this same original batch served as the source material for all subsequent accelerated and long-term stability sampling.

### 2.3. Conditions of Stability Investigations

Long-term stability: Samples were withdrawn from the original spray-dried batch described in Section 2.2 and were not independently remanufactured for this arm of the study. Powder samples equivalent to a 10 mg dose of THN, calculated from the drug content of each formulation, were filled into size 3 HPMC capsules [27]. The capsules were placed in tightly sealed glass containers and enclosed in polyethylene bags. Samples were stored in a desiccator over fresh silica gel, corresponding to an approximate relative humidity of 20–30% RH at room temperature 25 ± 2 °C. After 1 year of storage, samples were withdrawn and characterized (hereafter, 1 year). Stability was evaluated under long-term low-humidity conditions to approximate the moisture protection afforded by high-barrier primary packaging under practical storage conditions.

Accelerated stability: Samples were withdrawn from the original spray-dried batch described in Section 2.2 and were not independently remanufactured for this arm of the study. Accelerated stability testing was conducted for 3 months in accordance with ICH guidelines [37]. Powder samples equivalent to a 10 mg THN dose were filled into size 3 HPMC capsules. For each formulation, a single glass container containing approximately 10 capsules was prepared for each sampling time point and sealed within a soft multilayer polyethylene bag. Samples were stored in a calibrated constant climate chamber (Binder KBF 240; Binder GmbH, Tuttlingen, Germany) at 40 ± 2 °C and 75 ± 5% RH [30]. The chamber continuously controlled and monitored temperature and RH and the measured RH represents the external storage environment, while the actual RH inside the packaging system was not directly monitored. Containers were withdrawn after 1 and 3 months (hereafter, 1 mon and 3 mon), and freshly prepared samples (0 mon) served as the baseline. At each time point, the powders were characterized using the analytical methods described below.

### 2.4. Particle Size Distribution Investigation by Laser Diffraction

Particle size distributions were determined by dry laser diffraction using a Malvern Mastersizer Scirocco 2000 (Malvern Instruments Ltd., Malvern, Worcestershire, UK). Samples were analyzed at an air pressure of 2.0 bar using the refractive index values provided in the Malvern database, a measurement time of 12 s, and a vibration-feed setting of 75%. The evaluated parameters were D [0.1], D [0.5], D [0.9], span (calculated using Equation (1)), specific surface area (SSA), surface-weighted mean diameter (D [2,3]), and volume-weighted mean diameter (D [4,3]).

Span = (D [0.9] − D [0.1])/D [0.5](1)

### 2.5. Particle Morphology Assessment by Scanning Electron Microscopy (SEM)

Particle morphology was examined by scanning electron microscopy (SEM; Hitachi S-4700, Hitachi Scientific Ltd., Tokyo, Japan). Samples were coated with gold–palladium for 90 s under argon at high vacuum using a Bio-Rad SC 502 sputter coater (VG Microtech, Uckfield, UK). Imaging was performed using the operating parameters reported in our previous studies [33,36]: a chamber pressure of 1.3–13.0 mPa, an accelerating voltage of 10 kV, and a beam current of 10 nA.

### 2.6. X-Ray Powder Diffraction (XRPD) for Structural Investigation

XRPD patterns were recorded using a Bruker D8 Advance diffractometer equipped with a VANTEC-1 detector (Bruker AXS GmbH, Karlsruhe, Germany) and Cu Kα1 radiation (λ = 1.5406 Å). Finely ground samples were scanned from 3° to 40° 2θ at 40 kV and 40 mA, with a step size of 0.01° and a counting time of 0.1 s per step. The instrument was calibrated using an Al_2_O_3_ standard. Diffractograms were processed in OriginPro 8.5 (OriginLab Corporation, Northampton, MA, USA) and DIFFRAC.EVA V5.2 (Bruker AXS GmbH, Karlsruhe, Germany) using Kα2 correction, smoothing, and background subtraction. Crystallinity indices were calculated using Equation (2), where Xc is the crystallinity index and A_crystalline_ and A_total_ are the integrated areas of the crystalline peaks and the total diffractogram, respectively.

Xc (%) = (A_crystalline_/A_total_) × 100 (2)

### 2.7. Fourier-Transform Infrared (FTIR) Spectroscopy for Compatibility Investigation

FTIR spectra were recorded using a Bio-Rad Digilab FTS-65A/896 spectrometer (Philadelphia, PA, USA) over 4000–400 cm^−1^ at a resolution of 4 cm^−1^. Approximately 0.5 mg of sample was mixed with 22 mg of dry KBr and compressed into a disk under a 10-t load. Each sample was scanned 128 times. Spectra were smoothed, baseline-corrected, and normalized using OriginPro 8.5 (OriginLab Corporation, Northampton, MA, USA).

### 2.8. Thermal Analysis: Differential Scanning Calorimetry (DSC) and Thermogravimetric Analysis (TGA)

Thermal properties were evaluated by differential scanning calorimetry (DSC) using a Mettler Toledo DSC 821e instrument (Schwerzenbach, Switzerland). Samples (3–5 mg) were sealed in airtight aluminum pans and heated from 25 to 300 °C at 5 °C/min under an argon purge of 10 L/h; an empty purged pan served as the reference. The instrument was calibrated with indium according to the manufacturer’s instructions, and melting points were determined using STARe SW 16.30 software. Residual moisture, thermal stability, and decomposition behavior were assessed by thermogravimetric analysis (TGA) using a Mettler Toledo TG 821e instrument with STARe V9.1 software. Samples (approximately 20 mg) were placed in aluminum pans and heated from 30 to 300 °C at 10 °C/min under dry nitrogen at 100 mL/min. Weight loss profiles were evaluated from normalized thermograms.

### 2.9. Aerodynamic Performance Through the Andersen Cascade Impactor (ACI)

In vitro aerodynamic performance was assessed using an Andersen cascade impactor (ACI; Apparatus D, Copley Scientific Ltd., Nottingham, UK), as specified by the European Pharmacopoeia [38]. A flow rate of 60 L/min was applied for 4 s to draw a total volume of 4 L. The system comprised an HCP5 high-capacity pump, a TPK critical-flow controller, and a DFM 2000 mass-flow meter (Copley Scientific Ltd.). The ACI stages were coated with a thin layer of 1% Span 80 in cyclohexane to reduce particle bounce and approximate the adhesive properties of airway surfaces. Powders were aerosolized using a Breezhaler^®^ device (Novartis International AG, Basel, Switzerland). After actuation, the inhaler and capsule (Inh), mouthpiece adaptor (MA), USP induction port (IP), ACI stages (−1 to 6), and filter (F) were rinsed with 10% ethanol to recover deposited THN. THN was quantified at 272 nm using an ATI UNICAM UV/Vis spectrophotometer (Cambridge, UK) after appropriate dilution. The calibration equation was y = 52.849x + 0.097 (R^2^ = 0.9964). Aerodynamic parameters, including emitted fraction (EF; Equation (3)), fine particle fraction below 5 µm (FPF < 5 µm; Equation (4)), and mass median aerodynamic diameter (MMAD), were calculated using Inhalytix™ software (version 2.0.6; Copley Scientific Ltd., Nottingham, UK). All measurements were performed in triplicate.

EF% = [(initial mass − final mass remaining in capsule)/(initial mass)] × 100(3)

FPF% = [(mass of fine particles (<5 μm))/(total emitted mass)] × 100(4)

### 2.10. Cytotoxicity Investigation on the A549 Cell Line

The effects of THN-RAF and its individual components on A549 cell viability were assessed using the standard MTT [3-(4,5-dimethylthiazol-2-yl)-2,5-diphenyltetrazolium bromide] assay as described in our previous work where THN-TRE was tested [33]. A549 human alveolar basal epithelial cells (ATCC^®^, Manassas, VA, USA) were seeded in 96-well plates at 4 × 10^4^ cells per well in 100 µL of minimal essential medium containing Earle’s salts or RPMI medium. The medium was supplemented with 10% heat-inactivated fetal bovine serum, 2 mM L-glutamine, 1× nonessential amino acids, 4 mM HEPES, and 25 µg/mL gentamicin. Before treatment, cells were incubated for 24 h at 37 °C in a humidified atmosphere containing 5% CO_2_. The medium was then replaced with fresh medium containing two-fold serial dilutions of the test formulation from 500 to 0.98 µg/mL; untreated cells receiving culture medium alone served as controls. After 24 h of exposure, 20 µL of MTT solution (5 mg/mL) was added to each well, and the plates were incubated for 4 h at 37 °C. Formazan crystals were solubilized by adding 100 µL of 10% sodium dodecyl sulfate in 0.01 M HCl, followed by overnight incubation at 37 °C in 5% CO_2_. Optical density was measured at 570/650 nm using an EZ Read 400 ELISA reader (Biochrom, Cambridge, UK). Cell viability was expressed relative to the untreated control and calculated using Equation (5). Each sample was tested in quadruplicate.

Cell viability (%) = (absorbance of sample/absorbance of control) × 100(5)

The selected concentration range encompassed the estimated pulmonary exposure after inhalation, based on the nominal dose (10 mg), the formulation FPF (approximately 40–50%), and the reported lung lining fluid volume (10–70 mL) [39]. The range was extended beyond the estimated exposure levels to characterize the concentration–response relationship and identify potential concentration-dependent cytotoxicity [40].

### 2.11. Statistical Analysis

Data were analyzed using GraphPad Prism 10.1.2 (GraphPad Software, San Diego, CA, USA). For laser diffraction (*n* = 3), ACI (*n* = 3), and the MTT assay (*n* = 4), each n represents an independent sub-sample from the batch aliquot withdrawn at each time point, analyzed as a separate technical replicate. One-way ANOVA with Tukey’s post hoc test was used for multiple comparisons across storage time points. Values are presented as mean ± SD, with significance set at *p* ≤ 0.05 (* *p* ≤ 0.05; ** *p* ≤ 0.01; *** *p* ≤ 0.001). XRPD, FTIR, DSC, and TGA were performed as single determinations per sample per time point.

## 3. Results

### 3.1. Particle Size Distribution Results

Laser-diffraction analysis (Table 1) showed that both co-spray-dried formulations remained within the low-micrometer range (D [0.5], 3.88–6.41 µm) across the tested time points. The fresh samples had comparable span values (2.69 for THN-RAF and 2.08 for THN-TRE), indicating moderately broad particle size distributions. Under accelerated conditions, THN-RAF showed only limited particle growth, whereas THN-TRE exhibited a marked increase in particle size after 3 months, most likely because of moisture-induced agglomeration [41]. One-way ANOVA confirmed that this increase was statistically significant for both D [0.5] and D [0.9] at 3 months relative to all other time points (*p* < 0.0001 for THN-TRE), whereas the corresponding increase for THN-RAF, though smaller in magnitude, was also statistically significant for D [0.5] (*p* < 0.001). Particle size after 1 year of desiccator storage was not significantly different from their initial values for either formulation, statistically supporting the preservation of particle-size distribution under long-term low-humidity conditions. Overall, RAF provided greater protection against stress-induced particle growth than TRE. Because physical particle size measured by laser diffraction influences aerodynamic particle size [42], these findings were considered together with the aerodynamic results presented in Section 3.7.

### 3.2. Morphology of the Particles

**Figure 1:**
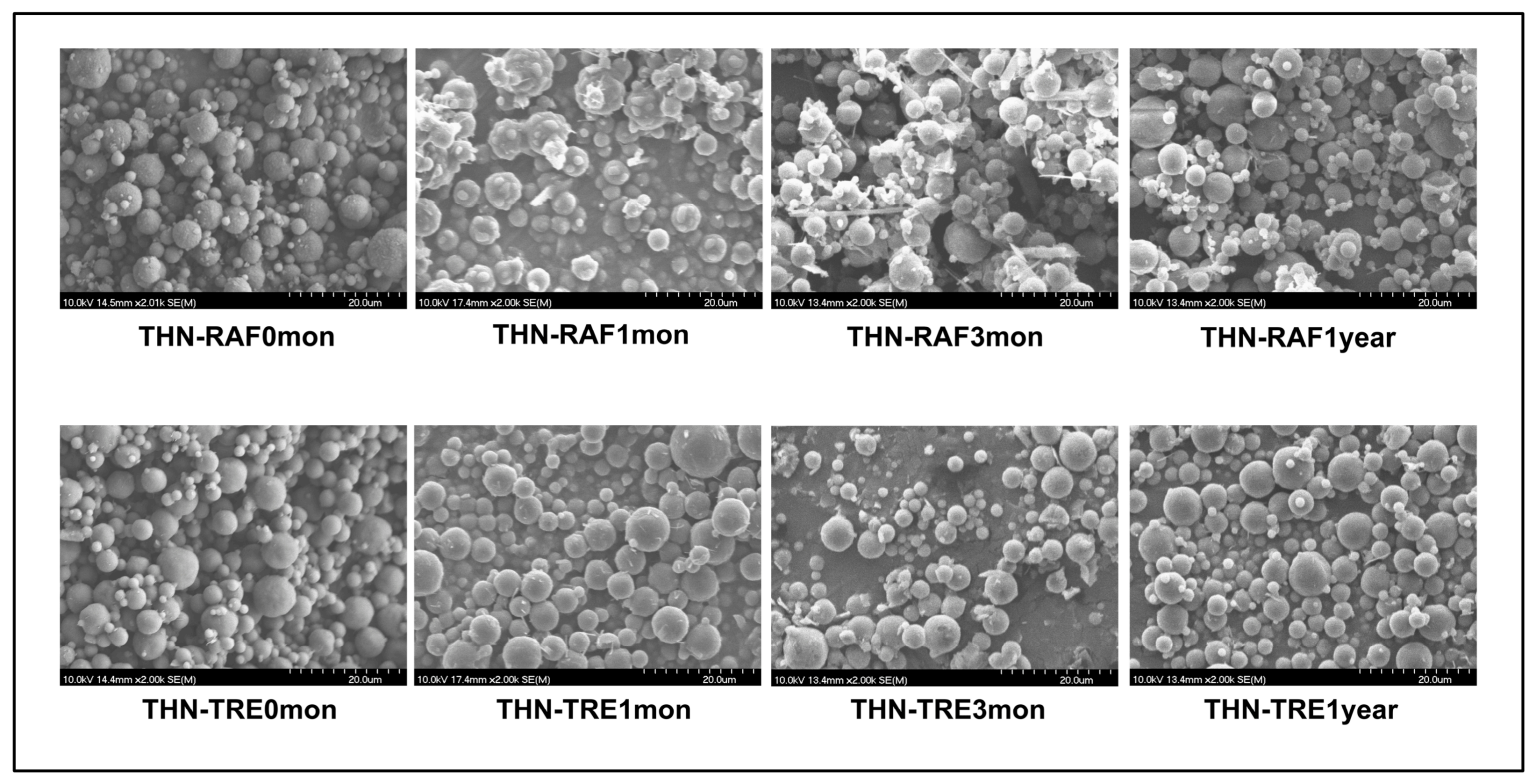
Temporal changes in morphology and particle size of the optimized formulations characterized by scanning electron microscopy (SEM).

The SEM images in Figure 1 show time-dependent morphological changes in both optimized co-spray-dried formulations. Fresh samples consisted of smooth, spherical microparticles, a morphology typical of spray-dried powders [43]. Under accelerated storage, both formulations exhibited progressive surface and morphological changes that were detectable after 1 month and more pronounced after 3 months. These changes included particle fusion, surface irregularities, and features consistent with recrystallization, such as crystalline bridges and outgrowths. THN-TRE showed less clustering than THN-RAF in the SEM images, despite its larger laser-diffraction particle size after 3 months. This could be attributed to the fact that SEM captures only localized morphology, whereas laser diffraction reflects the bulk volume-weighted distribution and is therefore more sensitive to infrequent large agglomerates. Previous work has shown that HPMC capsules minimize morphological changes and agglomeration under accelerated conditions [27]. Another study reported that leucine alone limited surface alteration and agglomeration during storage, whereas formulations containing polyvinyl alcohol or cyclodextrin showed visible aggregation [44]. By contrast, both formulations remained largely unchanged after long-term desiccator storage and retained their initial morphology. These observations indicate that DPI composition strongly influences morphological stability during storage.

### 3.3. XRPD Patterns and Crystallinity

**Figure 2:**
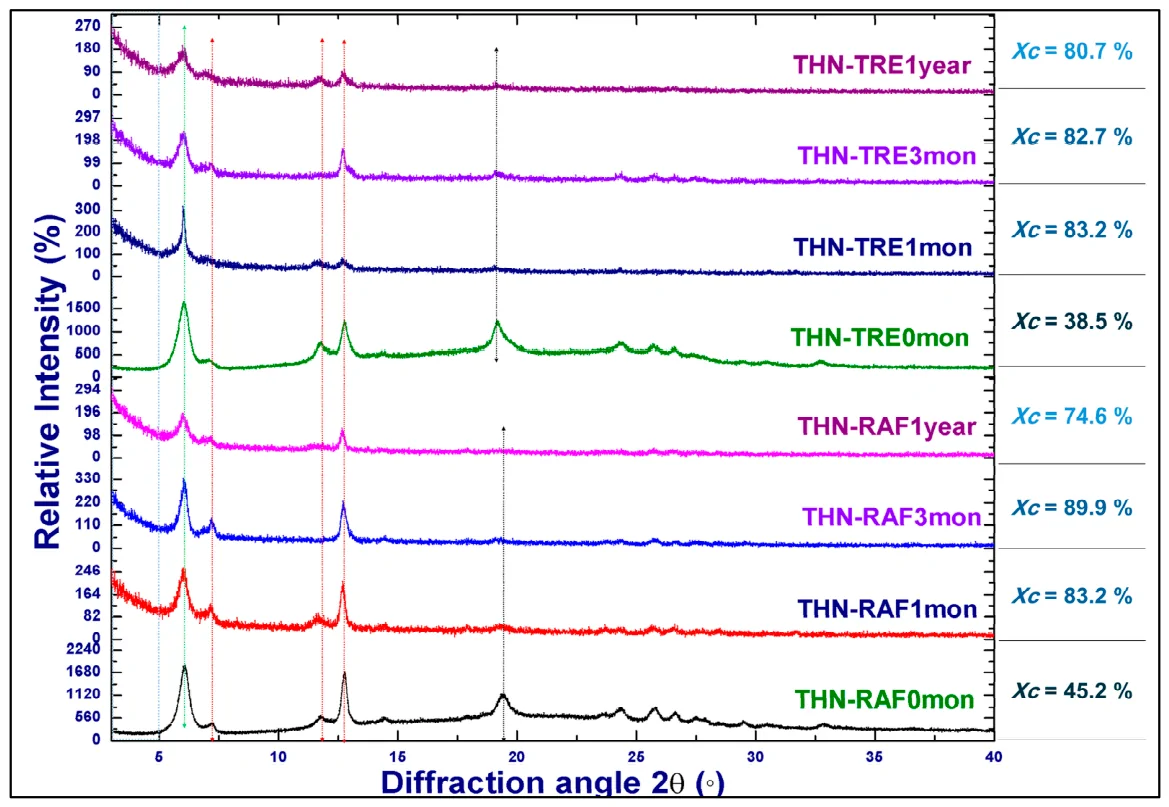
X-ray powder diffraction (XRPD) patterns of the optimized formulations showing time-dependent changes in crystallinity (Xc, crystallinity index).

The solid-state properties of THN-RAF and THN-TRE after accelerated and long-term storage were assessed by XRPD, and the corresponding crystallinity indices (Xc) are shown in Figure 2. Fresh THN-RAF and THN-TRE samples were partially amorphous, with Xc values of 45.2% and 38.5%, respectively, as expected for spray-dried powders; nevertheless, characteristic THN, LEU, and GLY peaks remained evident, as described previously [33,36]. Under accelerated conditions, crystallinity increased substantially within 1 month and reached 89.9% for THN-RAF and 82.7% for THN-TRE after 3 months, indicating formulation-specific recrystallization behavior. After long-term storage, Xc values were lower than the maximum values observed under accelerated conditions but remained higher than those of the fresh samples (74.6% and 80.7% for THN-RAF and THN-TRE, respectively). These findings are consistent with reports that amorphous spray-dried TRE progressively recrystallizes as temperature and RH increase [45]. The composition of spray-dried inhalation powders is also known to influence both initial crystallinity and the propensity for recrystallization under stress conditions [44].

### 3.4. FTIR Spectra and Chemical Integrity

**Figure 3:**
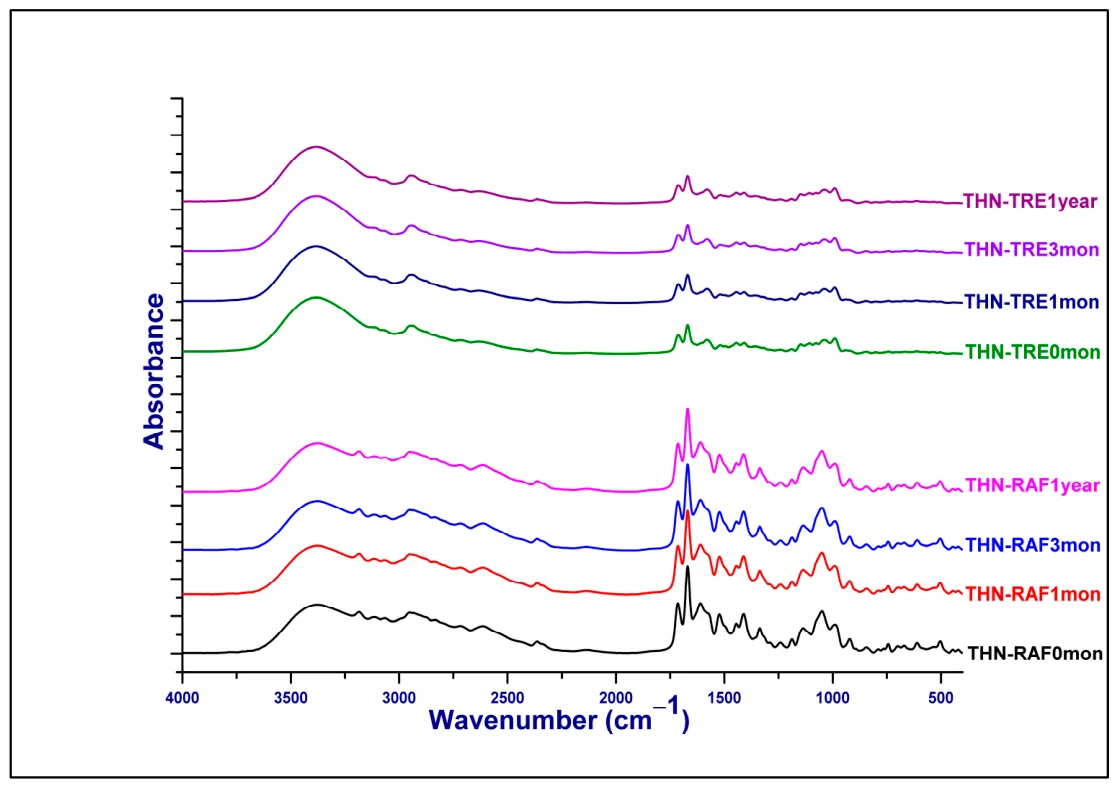
Fourier-transform infrared (FTIR) spectra of the optimized formulations illustrating temporal changes in molecular interactions and component compatibility.

The FTIR spectra (Figure 3) support the chemical integrity of THN and provide complementary evidence for the solid-state changes detected by XRPD and DSC. Across all time points and both formulations, the THN carbonyl/imidazole absorption at approximately 1670 cm^−1^ remained unshifted, and no new bands or peak splitting appeared, indicating the absence of degradation or drug–excipient interaction throughout storage. The broad O–H/N–H stretching envelope (3300–3600 cm^−1^), attributed to hydrogen-bonded hydroxyl groups of the amorphous matrix, remained unchanged in position and width at all time points for both formulations, suggesting no detectable alteration in hydrogen-bonding environment despite the concurrent rise in crystallinity. The 1500–1750 cm^−1^ and 950–1200 cm^−1^ regions, reflecting amide/carboxylate and saccharide skeletal (C–O–C, C–C) vibrations, showed slight changes with storage. For both formulations, slight sharpening in these regions increased gradually, paralleling the rise in Xc (Section 3.3). Because FTIR reflects local molecular order rather than long-range crystalline periodicity, these minor intensity changes, without any accompanying frequency shifts, could be considered consistent with the partial recrystallization occurring without chemical alteration of the drug or excipients. Taken together with the preserved carbonyl position and the supporting thermal (Section 3.5) and diffraction (Section 3.3) data, these results suggest that the observed instability during storage was physical rather than chemical in nature.

### 3.5. DSC Thermograms and Thermal Behavior

**Figure 4:**
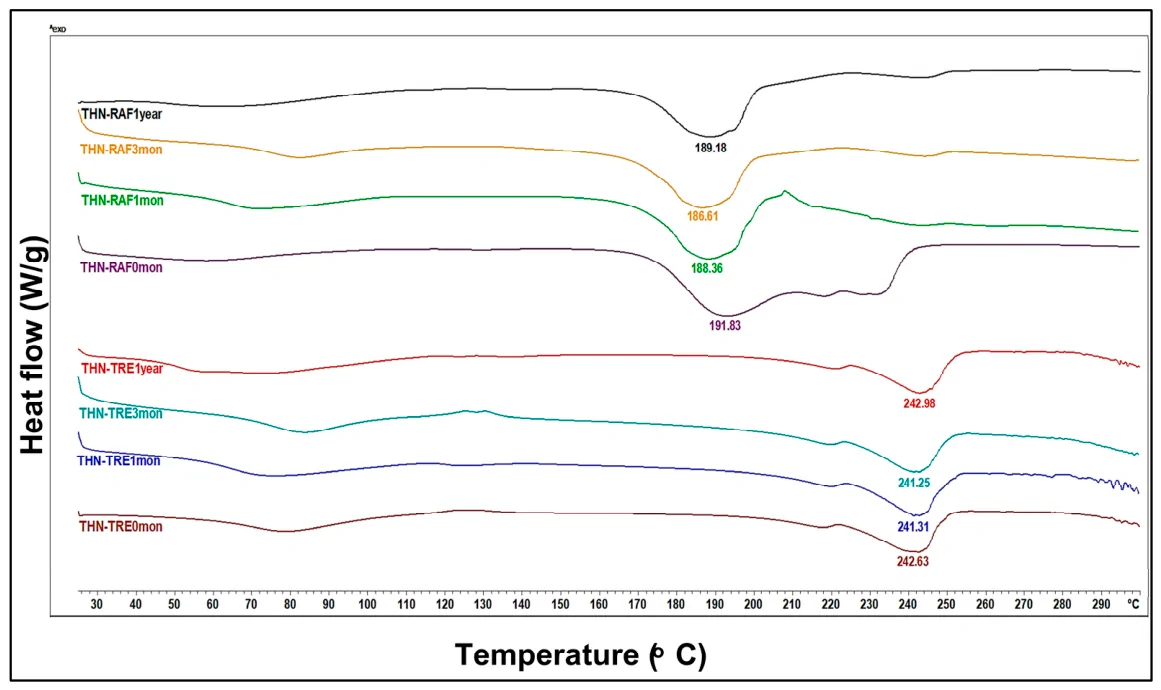
Differential scanning calorimetry (DSC) thermograms of the optimized formulations indicating time-dependent thermal behavior and solid-state transitions.

DSC measurements assess the potential for phase transitions or polymorphic shifts during storage, interpreted here alongside the crystallinity indices (Xc) obtained by XRPD (Section 3.3, Figure 2). The thermograms of THN-RAF and THN-TRE are presented in Figure 4; no discrete glass-transition preceded the main endotherm in either series, likely because the heating rate (5 °C/min) does not resolve a Tg overlapping with moisture-related effects below 100 °C [46]. For THN-RAF, the single endotherm observed is attributed to the melting of crystalline THN formed within the matrix during storage; this endotherm appeared around 187–192 °C and shifted slightly from 191.83 °C (0 mon) to 188.36 °C (1 mon), 186.61 °C (3 mon), and 189.18 °C (1 year); a pattern that broadly varied inversely with the crystallinity index, which rose from 45.2% to a maximum of 89.9% at 3 months under stress conditions and 74.6% at 1 year in desiccator, with the lowest melting temperatures coinciding with the highest Xc. For THN-TRE, a distinct thermal event appeared at a consistently higher temperature range (241–243 °C) across all time points, with nearly superimposable thermograms. This event is tentatively attributed to a solid-state transition associated with crystalline THN within the TRE-based matrix; however, as DSC was a single determination per time point without confirmatory techniques, this assignment, and any inference of a distinct local crystalline environment relative to THN-RAF, remains uncertain and requires further investigation. The stable position of this thermal event for THN-TRE aligns with the XRPD data, where Xc rose to approximately 83% within the first month under accelerated conditions and remained similar (80.7%) after 1 year under desiccator storage, indicating that THN-TRE reached a stable crystalline state more rapidly. Both formulations nonetheless reached comparably high crystallinity by 1 year (74.6% and 80.7%) with no degradation-related exothermic events, suggesting overall thermal robustness in both systems.

### 3.6. TGA Thermograms, Moisture Content, and Decomposition

**Figure 5:**
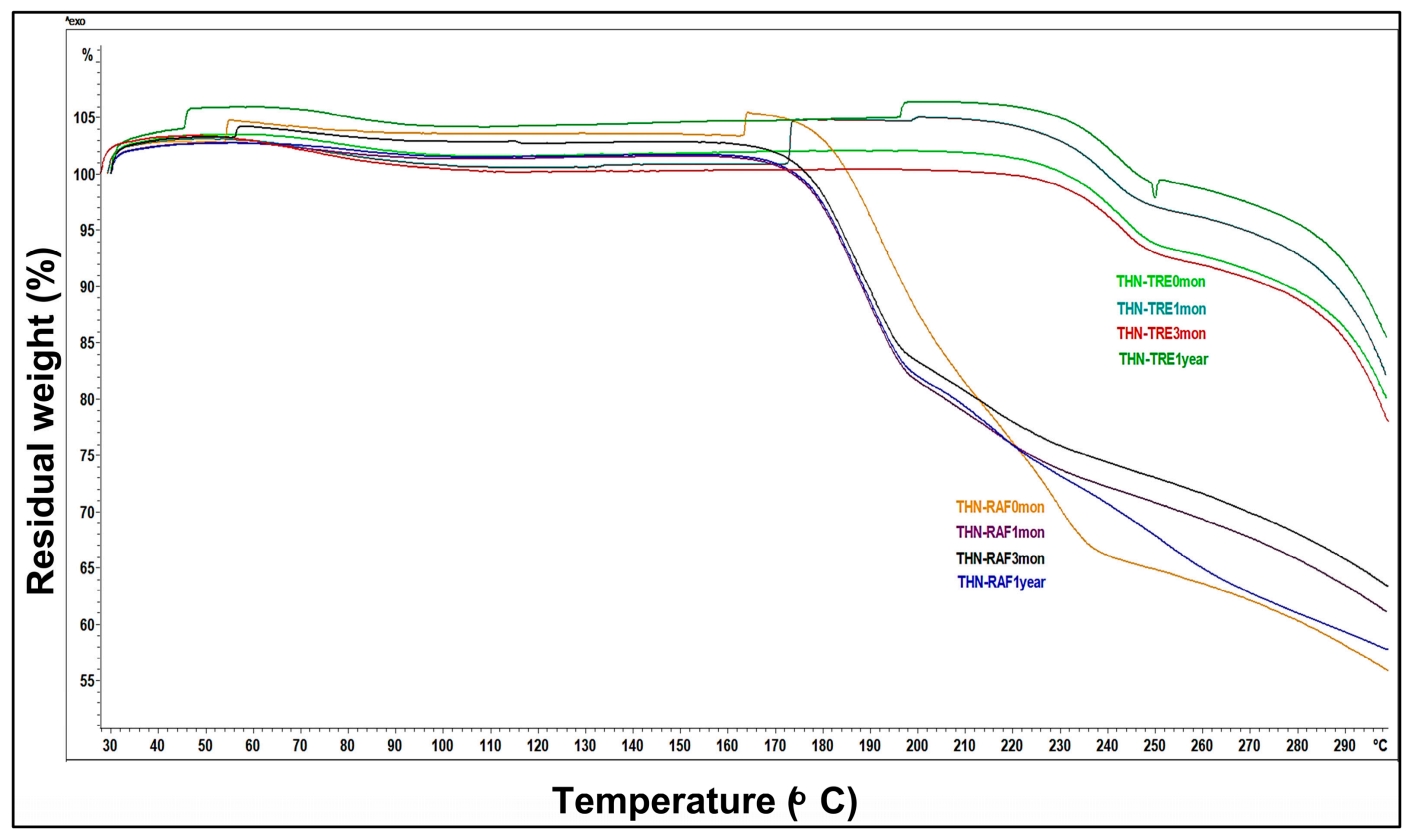
Thermogravimetric analysis (TGA) curves of the optimized formulations demonstrating thermal stability and degradation profiles over time.

TGA was conducted on THN-TRE and THN-RAF spray-dried powders at all stability withdrawal time points, as shown in Figure 5. Each thermogram showed a minor initial weight loss below 100 °C, attributed to residual surface and hydrate-associated moisture, followed by a single major degradation step. For THN-RAF, this step began consistently around 170–180 °C, with residual mass at 300 °C ranging between 55% and 65%. For THN-TRE, degradation began at a higher temperature, above 220 °C, with residual mass around 75–85%; this higher onset likely reflects the greater thermal stability of the TRE-based matrix relative to RAF. For both formulations, the degradation profile and residual mass remained comparable across fresh and stored samples, with no additional degradation steps or unexpected weight loss detected at any time point, potentially indicating preserved chemical integrity under both storage conditions. It should be noted that TGA reflects moisture content only at the time of measurement, not moisture uptake during storage; the stable initial weight-loss step therefore does not exclude moisture-driven plasticization as a contributor to the recrystallization and agglomeration observed by XRPD and laser diffraction (Section 3.1, Section 3.2 and Section 3.3). Overall, these results could indicate satisfactory thermal and chemical stability for both formulations, with the observed physical changes occurring largely independently of detectable degradation.

### 3.7. In Vitro Aerodynamic Properties

**Figure 6:**
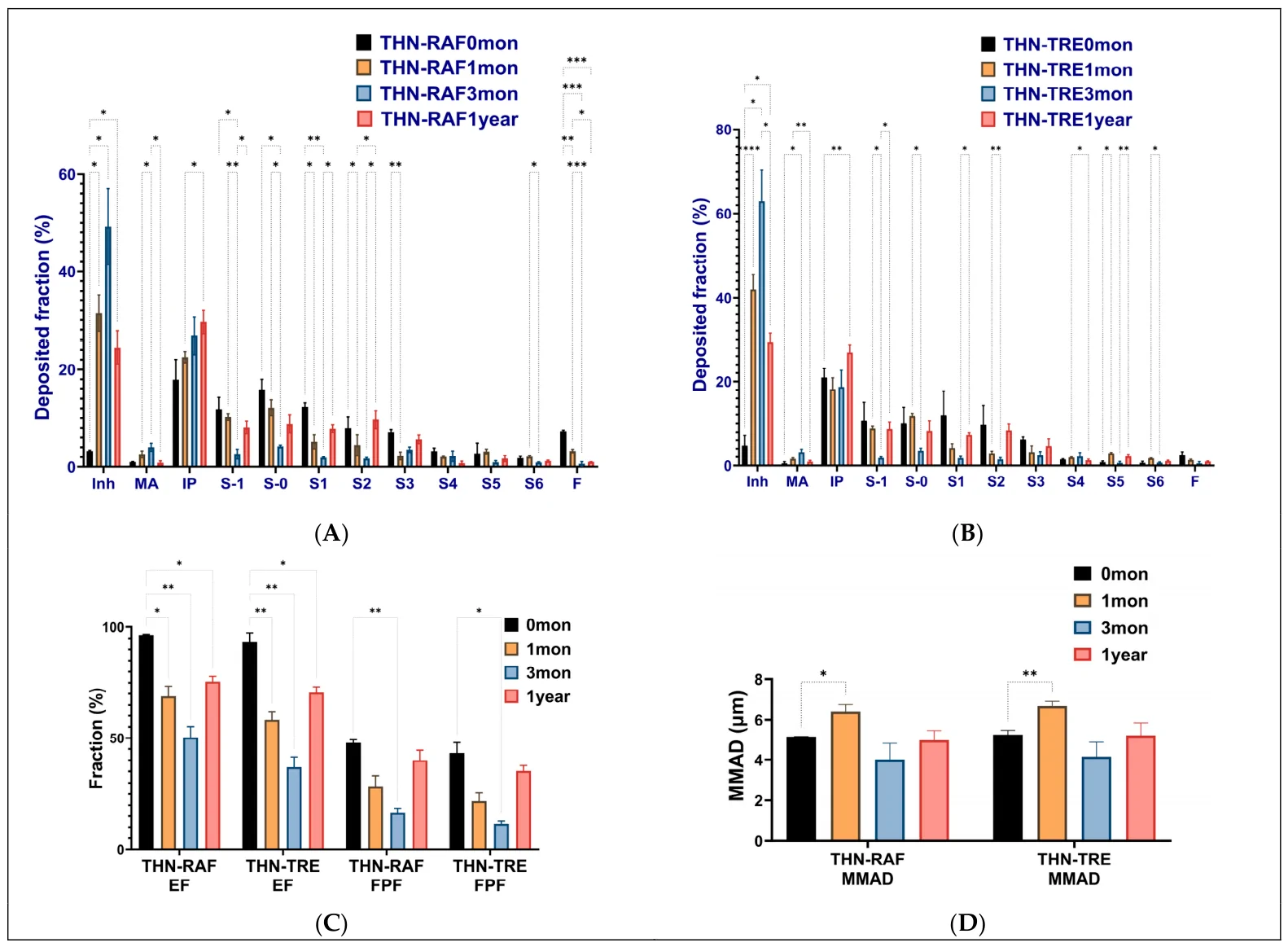
In vitro aerodynamic outcomes measured by Andersen cascade impactor (ACI) (average ± SD, *n* = 3, * *p* ≤ 0.05; ** *p* ≤ 0.01; *** *p* ≤ 0.001). (**A**): drug deposition of THN-RAF, (**B**): drug deposition of THN-TRE, (**C**): emitted fraction (EF) and fine particle fraction (FPF), (**D**): mass median aerodynamic diameter (MMAD). Abbreviations: Inh: Inhalation device; MA: mouthpiece adaptor; IP: induction port; S: ACI stage; F: filter.

Figure 6A–D illustrates the in vitro aerodynamic performance of both leading formulations, THN-RAF and THN-TRE, throughout the stability assessment, measured via ACI. At the initial point, both optimized powders achieved high EFs of 93.62–96.45% and FPFs of 43.23–47.84% (Figure 6C), supporting efficient lung deposition, with geometric standard deviations of 2.02–2.72 indicating an approximately monomodal aerodynamic particle-size distribution beneficial for reducing delivered-dose variability [47].

The stage-by-stage deposition profiles (Figure 6A,B) help explain these summary parameters. At the initial time point (0 mon), deposited fraction was distributed relatively evenly across the lower ACI stages (S1–F), which represent the fine, respirable fraction, with only a minor fraction retained in the device (Inh). Under accelerated storage, this pattern shifted progressively: the fraction retained in the device increased markedly, reaching its maximum at 3 months for both formulations (THN-RAF: 49.23%; THN-TRE: 62.95%), while deposition on the lower stages (S1–F) declined correspondingly. This redistribution toward the device, rather than a uniform loss of drug across all stages, appears to be the main driver of the reduced EF and FPF values observed under stress conditions (Figure 6C). The induction port (IP) fraction remained comparatively stable across time points and formulations, suggesting that upper-stage deposition, representing coarser or agglomerated particles, was less affected than the fine-particle stages. THN-TRE showed a somewhat greater increase in device retention at 3 months than THN-RAF, consistent with its more pronounced particle agglomeration described in Section 3.1. Device retention for both formulations after one year of desiccator storage was lower than the corresponding retention measured under stress conditions, though it remained slightly above the 0 mon baseline, paralleling the partial preservation in EF and FPF values (Figure 6C). This trend is consistent with a previous study reporting FPF deterioration under high RH (≥75%) but little change under lower RH (≤55%) [48], and aligns with our long-term aerodynamic outcomes, where the decrease in FPF relative to fresh samples was comparatively small, although a slight decrease in EF remained noticeable.

MMAD values (Figure 6D), by contrast, remained close to 5 µm at most time points, near the upper limit considered favorable for effective aerosolization, and showed smaller relative changes than EF and FPF; a transient increase was observed at 1 month for both formulations. This pattern is consistent with a previous study on the relevance of FPF and MMAD in estimating pulmonary drug deposition, which concluded that FPF better reflects total lung deposition, whereas MMAD is more indicative of the regional distribution of the deposited dose [49].

### 3.8. In Vitro Cytotoxic Effects on the A549 Cells

**Figure 7:**
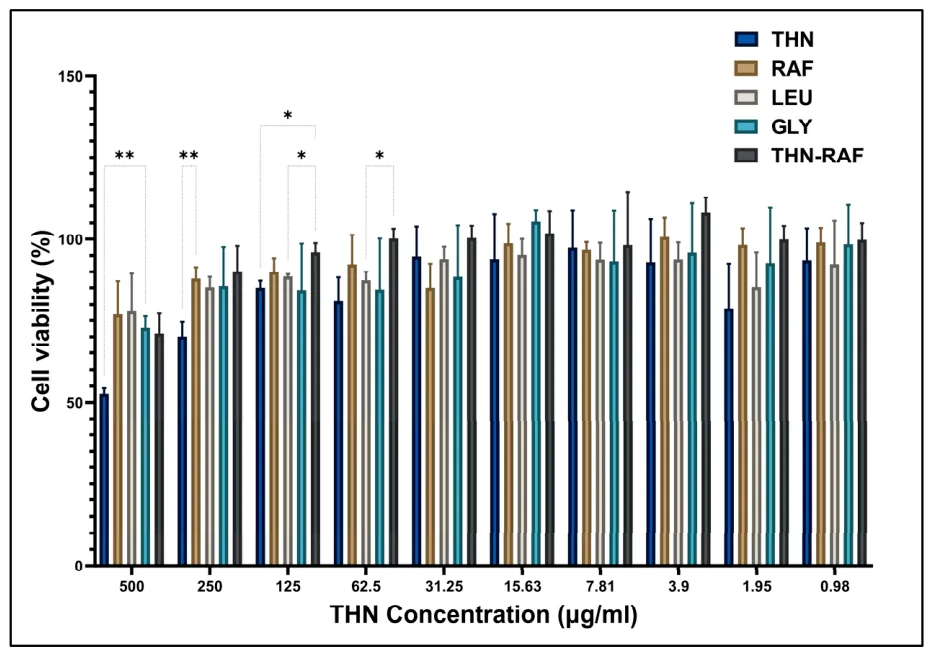
Cell viability of A549 alveolar epithelial cells after 24 h treatment with the raw components and the optimized formulation THN-RAF measured by MTT assay. THN, theophylline; RAF, raffinose; LEU, l-leucine; GLY, glycine. Data are presented as mean ± SD (*n* = 4). * *p* ≤ 0.05 and ** *p* ≤ 0.01 indicate significant differences compared to the untreated control.

A549 cell viability after exposure to the tested materials is shown in Figure 7. THN-RAF maintained cell viability above 70% at all tested concentrations (0.98–500 µg/mL). RAF, LEU, and GLY likewise maintained viability above 70% throughout the tested range. Raw THN produced the greatest reduction in viability at the highest concentrations and yielded significantly lower viability than THN-RAF at equivalent THN concentrations. Incorporation of THN into the co-spray-dried matrix therefore reduced its apparent cytotoxic effect under the tested conditions. Overall, THN-RAF showed favorable preliminary cytocompatibility in A549 cells at concentrations that encompassed and exceeded the estimated pulmonary exposure range, which is consistent with our previous outcomes regarding THN-TRE [33].

### 3.9. Comparative Summary of Stability Outcomes

Table 2 summarizes the principal physicochemical and aerodynamic findings for THN-RAF and THN-TRE across all storage conditions.

## 4. Discussion

Unlike our previous papers, which focused on formulation development and initial characterization [33,36], the present study investigates how storage under different environmental conditions influences the stability and performance of our optimized DPI formulations.

The difference between the accelerated and long-term storage results can be explained by the behavior of amorphous saccharide matrices under different conditions (temperature and relative humidity). In glassy systems, physical stability depends mainly on how close the storage temperature is to the glass transition temperature (Tg), which itself depends strongly on moisture content [46]. At 40 °C/75% RH, water uptake likely plasticized saccharide matrices, lowering their Tg below the storage temperature and inducing a glass-to-rubbery state transition. Under these conditions, increased molecular mobility would facilitate crystal nucleation and growth, thereby promoting physical instability and agglomeration [50,51], which could lead to aerodynamic performance deterioration.

Under desiccator storage (25 ± 2 °C, low RH), the powders likely remained in a glassy state with limited molecular mobility, thereby preserving particle morphology and aerodynamic performance despite the extended storage period. In contrast, exposure to elevated humidity can promote water sorption, plasticization, and increased molecular mobility, accelerating crystallization and agglomeration processes. The preservation of performance after one year of desiccator storage, despite the substantially longer storage period, suggests that humidity-driven mobility, rather than storage time alone, is the dominant factor governing the physical stability of amorphous inhalable powders [52].

It should be noted that the comparison between long-term desiccator storage and accelerated storage (40 °C/75% RH) has inherent limitations because both storage temperature and relative humidity differ simultaneously. Consequently, the observed changes cannot be attributed exclusively to either thermal or moisture effects but instead reflect the combined influence of these environmental stressors. The purpose of this comparison was therefore not to determine the independent contribution of each variable, but rather to assess formulation robustness under two practically relevant storage scenarios representing optimal low-moisture storage and accelerated stress conditions.

Importantly, the applied long-term desiccator storage does not correspond to the standard ICH long-term condition of 25 °C/60% RH. The desiccator condition was intentionally selected to evaluate the intrinsic behavior of these moisture-sensitive amorphous powders under protective low-humidity conditions.

The greater resistance to stress-induced agglomeration observed for THN-RAF may be attributed to differences in the molecular structure between RAF and TRE, since differences in saccharide structure can influence hydrogen bonding, molecular mobility, and the stability of amorphous matrices [53], which may contribute to the enhanced resistance of THN-RAF to moisture-induced destabilization. Although differences in excipient composition may have influenced particle characteristics, the superior stability of THN-RAF could be primarily associated with the RAF-based matrix, suggesting that matrix composition rather than the presence of GLY was the dominant factor governing the observed resistance to moisture-induced destabilization.

The decline in aerodynamic performance under stress conditions is consistent with the established relationship between particle agglomeration and aerosolization efficiency in DPIs. Although both formulations exhibited substantial recrystallization during storage, THN-TRE showed a markedly greater increase in particle size, indicating that recrystallization was associated with more extensive particle growth and agglomeration. Such changes increase interparticle cohesion and reduce the ability of particles to disperse into the respirable size range during inhalation, resulting in lower FPF values. The concurrent increase in particle size (specifically D [0.9]) and decrease in FPF observed for THN-TRE support this interpretation, whereas the relatively stable particle size distribution and aerodynamic performance of THN-RAF suggest that its recrystallization pathway was less disruptive to particle dispersibility and lung-deposition potential [22,54].

The pronounced sensitivity of the formulations to elevated humidity also underscores the importance of packaging considerations during product development [55]. Accelerated testing of the present packaging configuration may not reflect the protection achievable with optimized commercial packaging. The favorable performance under desiccator storage suggests that maintaining a similarly low-humidity microenvironment could sustain formulation properties well beyond the tested timeframes. Achieving this in a marketed product would likely require high-moisture-barrier packaging, combined with low-humidity fill-finish operations. Capsule shell selection would also warrant consideration given its known influence on moisture exchange [25,26,27]. Such strategies could support longer shelf-life claims and reduce reliance on cold-chain storage. However, since desiccator conditions do not replicate the mechanical and handling stresses of commercial packaging, dedicated packaging studies would be needed to confirm these effects at scale.

It should also be noted that each formulation was produced as a single spray-dried batch, and the statistical replicates reported throughout this study reflect intra-batch technical and sub-sampling variability rather than inter-batch manufacturing reproducibility. Confirmation of batch-to-batch consistency was outside the scope of the present stability evaluation and should be addressed in future process-validation studies.

The considerable influence of humidity observed in this study highlights the limitations of assessing amorphous DPI stability using temperature-based accelerated testing alone. While the ICH Q1A(R2) accelerated condition (40 °C/75% RH) is valuable for identifying potential failure mechanisms, moisture-driven plasticization and Tg depression are often the primary determinants of physical stability in amorphous formulations. Consequently, characterization of moisture sorption behavior and Tg across a range of humidity conditions may provide a more formulation-relevant understanding of stability than a single accelerated storage condition. Such data could help identify critical humidity thresholds for physical destabilization and support the development of more predictive shelf-life models for amorphous inhalable powders.

The cytotoxicity results can also be viewed in the context of the established use of saccharide- and amino acid-based excipients in pulmonary formulations [56,57,58,59]. Cell viability remained above 70% across the tested concentration range, indicating satisfactory in vitro biocompatibility. This finding is consistent with reports that many excipients used in pulmonary formulations exhibit cytotoxicity only at concentrations substantially higher than their approved inhalation levels, reflecting favorable safety margins [32].

The biological evaluation was limited to A549 cells, a widely accepted model for preliminary pulmonary epithelial cytocompatibility assessment. However, this model does not fully recapitulate the complexity of the respiratory tract. Accordingly, further studies using complementary pulmonary cell models, advanced air–liquid interface cultures, or in vivo models are warranted before broader conclusions regarding pulmonary safety can be made [60].

Together, these findings suggest that the principal development challenge for these THN-based DPIs is likely to be the maintenance of physical stability under humid conditions rather than acute in vitro cytotoxicity.

While the present findings provide strong evidence for humidity-driven physical destabilization, the underlying mechanisms were hypothesized from solid-state and morphological analyses rather than measured directly. Further studies using dynamic vapor sorption, humidity-dependent Tg measurements, and surface-sensitive analytical techniques could provide additional insight into the onset and spatial distribution of recrystallization. Evaluation of packaging moisture-barrier performance would also support the development of more predictive models for long-term storage stability.

## 5. Conclusions

This study provides a comprehensive stability and cytotoxicity characterization of saccharide-amino acid co-spray-dried THN DPI formulations. Long-term desiccator storage preserved the aerodynamic profiles of both formulations, yielding FPF values of approximately 40% and MMAD within the respirable range (4.99–5.21 µm), demonstrating the fundamental viability of both systems as stable DPI platforms and indicating that the observed increase in crystallinity did not compromise their key aerosol performance attributes. In contrast, accelerated conditions induced pronounced recrystallization (crystallinity indices approaching 90%), agglomeration particularly for the TRE-based formulation, which reached a D [0.9] of 217.08 µm, and aerodynamic deterioration (FPF as low as 11.55%). THN-RAF exhibited lower stress-induced agglomeration than THN-TRE, indicating greater resistance to structural changes during storage under stress conditions. This observation is consistent with reports that RAF can retard crystal growth and enhance the stability of amorphous systems. More broadly, saccharide molecular structure influences crystallization behavior and amorphous stability, which may explain the greater resistance of THN-RAF to agglomeration [61,62].

The pronounced difference between accelerated and desiccator storage outcomes indicates that moisture exposure is a critical determinant of physical stability for these amorphous formulations. It should be noted that the desiccator condition does not represent the standard ICH long-term storage condition; the present findings therefore primarily highlight formulation-specific moisture sensitivity rather than any inadequacy of ICH testing protocols.

Importantly, FTIR and TGA analyses suggested the preservation of chemical integrity under all storage conditions, which may indicate that the observed deterioration was primarily physical rather than chemical in nature. These findings could highlight the importance of controlling moisture-driven changes in particle morphology and solid-state stability to help maintain the performance of amorphous inhalable powders during storage.

THN-RAF demonstrated favorable cytocompatibility in A549 pulmonary epithelial cells, complementing the favorable biocompatibility profile previously reported for THN-TRE and supporting the suitability of this platform for pulmonary administration [33]. Although A549 cells are a well-established model for preliminary pulmonary toxicity assessment, the biological evaluation remains limited to a single epithelial cell line. Therefore, additional pulmonary cell models and more physiologically relevant systems are required before broader conclusions regarding pulmonary safety can be drawn [60]. Overall, our findings demonstrate that these formulations possess desirable in vitro characteristics for effective pulmonary therapy. These attributes support their potential for effective pulmonary delivery of THN when appropriately controlled storage conditions are maintained and justify their further preclinical development as inhalable therapeutic systems for pulmonary therapy; confirmation of pulmonary deposition and delivery consistency will require in vivo evaluation.

Future studies should investigate moisture sorption behavior, glass transition dynamics, and in vivo lung deposition to further elucidate the influence of environmental humidity on the long-term performance of amorphous inhalable powders, while incorporating intermediate stability sampling time points to enable degradation kinetic evaluation and more comprehensive characterization of formulation performance during storage.

## Figures and Tables

**Table 1:** Particle size distribution measurements obtained by laser diffraction.

Formulation	D [0.1] * (µm)	D [0.5] * (µm)	D [0.9] * (µm)	Span *	SSA * (m^2^/g)	D [2,3] * (µm)	D [4,3] * (µm)
THN-RAF0mon	1.82 ± 0.03	4.23 ± 0.09	13.23 ± 3.47	2.69 ± 0.77	1.69 ± 0.09	3.56 ± 0.18	7.17 ± 1.01
THN-RAF1mon	2.06 ± 0.14	4.10 ± 0.29	7.84 ± 0.67	1.41 ± 0.04	1.69 ± 0.13	3.57 ± 0.26	5.77 ± 2.31
THN-RAF3mon	2.62 ± 0.03	5.62 ± 0.06	12.86 ± 0.45	1.82 ± 0.10	1.24 ± 0.01	4.85 ± 0.02	16.10 ± 2.01
THN-RAF1year	1.74 ± 0.02	3.88 ± 0.12	8.21 ± 0.71	1.67 ± 0.14	1.87 ± 0.04	3.22 ± 0.07	6.15 ± 3.03
THN-TRE0mon	1.83 ± 0.37	4.31 ± 0.01	14.35 ± 7.20	2.08 ± 0.36	1.72 ± 0.17	3.51 ± 0.34	7.36 ± 1.84
THN-TRE1mon	2.00 ± 0.07	4.24 ± 0.12	8.76 ± 0.19	1.60 ± 0.06	1.66 ± 0.05	3.61 ± 0.11	7.38 ± 2.41
THN-TRE3mon	2.92 ± 0.12	6.41 ± 0.10	217.08 ± 45.97	33.42 ± 7.34	1.02 ± 0.00	5.87 ± 0.02	49.30 ± 12.49
THN-TRE1year	2.05 ± 0.12	4.26 ± 0.25	8.66 ± 1.18	1.55 ± 0.19	1.65 ± 0.11	3.65 ± 0.23	9.05 ± 5.01

* Results are expressed as average ± SD (*n* = 3). Statistical analysis (one-way ANOVA, Tukey’s post hoc test) was performed across storage time points for each formulation and parameter; significant differences (*p* ≤ 0.05) relative to the 0 mon baseline are indicated where applicable in the text.

**Table 2:** Comprehensive comparative summary of stability outcomes for co-spray-dried THN-RAF and THN-TRE dry powder inhaler formulations.

Analytical Parameter	Objective	THN-RAF Key Findings	THN-TRE Key Findings	Statistical Evidence	Pharmaceutical Significance
Particle size distribution (Laser diffraction)	Detect stress-induced particle growth/agglomeration	D [0.5]: 4.23→5.62 µm (3 mon)→3.88 µm (1 year) D [0.9]: 13.23→12.86 µm (3 mon); 8.21 µm (1 year)	D [0.5]: 4.31→6.41 µm (3 mon)→4.26 µm (1 year) D [0.9]: 14.35→217.08 µm (3 mon); 8.66 µm (1 year)	D [0.5] and D [0.9] increased at 3 mon vs. all other points: *p* < 0.0001 (TRE); D [0.5] *p* < 0.001 (RAF) No significant change vs. baseline after 1 year	RAF: more resistant to accelerated-stress agglomeration PSD preserved by low-humidity long-term storage
Particle morphology (SEM)	Visualize storage-induced surface/shape changes	Smooth, spherical fresh microparticles (0 mon) → progressive alterations, detectable at 1 mon and pronounced at 3 mon accelerated conditions Unchanged after 1 year desiccator storage	Same qualitative trend as THN-RAF Visibly less inter-particle clustering than RAF at 3 mon	Qualitative/Single determination per time point	Confirms that accelerated stress conditions, not only storage duration, drives particle fusion and surface recrystallization
Crystallinity index, Xc (XRPD)	Quantify amorphous-to-crystalline conversion during storage	45.2% (0 mon) → up to 89.9% (3 mon, accelerated) → 74.6% (1 year, desiccator)	38.5% (0 mon) → up to 83.2% (3 mon, accelerated) → 80.7% (1 year, desiccator)	Single determination per time point	Recrystallization under both conditions Long-term desiccator storage limits crystallinity gain, underscoring formulation moisture-sensitivity
Chemical integrity (FTIR)	Detect drug degradation or drug–excipient interaction	THN carbonyl/imidazole band (~1670 cm^−1^) unshifted at all time points No new bands or peak splitting	Same pattern as THN-RAF O–H/N–H envelope (3300–3600 cm^−1^) unchanged in position/width throughout	Single determination per time point	No spectroscopic evidence of chemical degradation within technique sensitivity
Thermal behavior (DSC)	Detect phase transitions/polymorphic shifts	THN melting endotherm shifts from 191.83 °C (0 mon) to 186.61 °C (3 mon, accelerated) and 189.18 °C (1 year) Shift tracks inversely with Xc	Thermal event tentatively associated with crystalline THN, stable at ~241–243 °C Nearly superimposable thermograms	Single determination per time point	TRE-based matrix: suggests more thermally stable local crystalline environment for THN No degradation-related exotherms
Residual moisture/decomposition (TGA)	Assess moisture content at measurement and thermal decomposition onset	Degradation onset ≈ 170–180 °C Residual mass at 300 °C = 55–65%	Degradation onset >220 °C Residual mass at 300 °C = 75–85%	Single determination per time point	Comparable degradation profile/residual mass across all storage time points No additional degradation steps detected
Aerodynamic performance (ACI)	Evaluate in vitro lung-deposition potential after storage	Baseline EF/FPF: 96.45%/47.84% Accelerated: device retention up to 49.23%; FPF as low as 16.4% 1-year desiccator: FPF ≈ 40%; MMAD = 4.99 µm	Baseline EF/FPF: 93.62%/43.23% Accelerated: device retention up to 62.95%; FPF as low as 11.55% 1-year desiccator: FPF ≈ 35%; MMAD = 5.2 µm	*n* = 3 per time point *p* < 0.05 (Figure 6)	Long-term desiccator storage preserves functional aerodynamic performance Accelerated stress is markedly deleterious, especially for THN-TRE, consistent with its greater agglomeration

Abbreviations: THN, anhydrous theophylline; RAF, raffinose; TRE, trehalose; LEU, L-leucine; GLY, glycine; PSD, particle size distribution; SEM, scanning electron microscopy; XRPD, X-ray powder diffraction; Xc, crystallinity index; FTIR, Fourier-transform infrared spectroscopy; DSC, differential scanning calorimetry; TGA, thermogravimetric analysis; ACI, Andersen cascade impactor; EF, emitted fraction; FPF, fine particle fraction (<5 µm); MMAD, mass median aerodynamic diameter; 0 mon/1 mon/3 mon, accelerated-storage time points (40 ± 2 °C/75 ± 5% RH); 0 mon/1 year, long-term desiccator storage time point (25 ± 2 °C, low RH).

## Data Availability

The original contributions presented in the study are included in the article; further inquiries can be directed to the corresponding author.

## Abbreviations

The following abbreviations are used in this manuscript:

A549	Human alveolar epithelial adenocarcinoma cell line
ACI	Andersen cascade impactor
ANOVA	Analysis of variance
Xc	Crystallinity index
D [0.1]	Particle diameter at the 10th percentile of the volume distribution
D [0.5]	Median particle diameter
D [0.9]	Particle diameter at the 90th percentile of the volume distribution
D [2,3]	Surface-weighted mean diameter
D [4,3]	Volume-weighted mean diameter
DPI	Dry powder inhaler
DSC	Differential scanning calorimetry
EF	Emitted fraction
FPF	Fine particle fraction
FTIR	Fourier-transform infrared spectroscopy
GLY	Glycine
HPMC	Hydroxypropyl methylcellulose
ICH	International Council for Harmonization of Technical Requirements for Pharmaceuticals for Human Use
LEU	Leucine
MMAD	Mass median aerodynamic diameter
RAF	Raffinose
RH	Relative humidity
SD	Standard deviation
SEM	Scanning electron microscopy
SSA	Specific surface area
Tg	Glass transition temperature
TGA	Thermogravimetric analysis
THN	Anhydrous theophylline
THN-RAF	Theophylline co-spray-dried with raffinose, leucine, and glycine
THN-TRE	Theophylline co-spray-dried with trehalose and leucine
TRE	Trehalose
XRPD	X-ray powder diffraction
